# Polypharmacy in Older Adults: A Narrative Review of Clinical Risks, Deprescribing Strategies, and Interdisciplinary Management

**DOI:** 10.3390/geriatrics11040096

**Published:** 2026-07-30

**Authors:** Himat Hussein Mamand, Nóra Rozmann, Miklós Sugár, Ahmed Lateef Abed Alkhaqani, Saya Hama, Bence Raposa

**Affiliations:** 1 Faculty of Health Sciences, University of Pécs, Vörösmarty Mihály Str. 4, 7621 Pécs, Hungary; mamand.hussein@edu.pte.hu; 2 Institute of Emergency Care, Pedagogy of Health and Nursing Sciences, Faculty of Health Sciences, University of Pécs, Vörösmarty Mihály Str. 4, 7621 Pécs, Hungary; nora.rozmann@etk.pte.hu (N.R.); miklos.sugar@etk.pte.hu (M.S.); 3 Doctoral School of Health Sciences, Faculty of Health Sciences, University of Pécs, Vörösmarty Mihály Str. 4, 7621 Pécs, Hungary; 4 College of Nursing, University of Raparin, Main Street, Ranya 46012, Iraq; 5 Institute of Basics of Health Sciences, Midwifery and Health Visiting, Faculty of Health Sciences, University of Pécs, Vörösmarty Mihály Str. 4, 7621 Pécs, Hungary

**Keywords:** polypharmacy, elderly, deprescribing, adverse drug reactions, medication review, STOPP/START, clinical decision support systems, interdisciplinary collaboration

## Abstract

**Background/Objective**: Polypharmacy, conventionally defined as the concurrent use of five or more medications, has emerged as a critical public health challenge in aging populations worldwide. Polypharmacy in older adults, driven by multimorbidity and age-related physiological changes, increases the risk of adverse drug reactions, drug interactions, falls, cognitive impairment, non-adherence, and preventable hospitalization. This study aims to provide a structured narrative synthesis of the current evidence on the incidence, risk factors, and clinical management of polypharmacy in elderly patients, with particular emphasis on deprescribing strategies, interdisciplinary care models, patient education, and digital clinical decision support technologies. **Methods**: A structured narrative literature review was conducted across PubMed, Scopus, and Web of Science using Boolean combinations of MeSH and free text terms including ‘polypharmacy,’ ‘elderly,’ ‘deprescribing,’ and ‘medication review.’ Eligible sources were peer-reviewed primary studies published between January 2016 and June 2026 that enrolled adults aged ≥65 years and reported validated prescribing review approaches, such as the STOPP/START criteria. The review drew on 40 primary studies spanning randomized controlled trials (RCTs) and observational, qualitative, and mixed-methods designs, which were interpreted and synthesized narratively across the principal thematic domains of polypharmacy management. **Results**: Polypharmacy was independently and consistently associated with DDIs, preventable hospitalizations, and functional decline across diverse clinical settings. Pharmacist-led medication reviews and interdisciplinary, team-based interventions produced the most robust improvements in prescribing appropriateness and meaningful reductions in potentially inappropriate medications (PIMs). Electronic clinical decision support systems (CDSSs) have demonstrated measurable benefits for safe deprescribing at scale, although usability limitations, incomplete workflow integration, and clinician resistance are significant implementation obstacles. **Conclusions**: Effective management of polypharmacy in older adults requires a multicomponent, patient-centered strategy that integrates evidence-based deprescribing algorithms, principally the STOPP/START criteria, sustained interdisciplinary collaboration, structured patient education, and technology-assisted decision support.

## 1. Introduction

Polypharmacy, most commonly operationalized as the concurrent use of five or more medications, represents one of the most pervasive and clinically consequential challenges confronting modern healthcare systems serving aging populations. Driven by demographic shifts towards an older global population and medical advances that successfully extend life expectancy, the volume of prescriptions issued per older adult has markedly increased over recent decades. Adults aged ≥ 65 years are disproportionately affected and frequently manage several coexisting long-term conditions, including cardiovascular disease, type 2 diabetes mellitus (T2DM), and chronic kidney disease (CKD), each typically governed by separate clinical practice guidelines. The cumulative application of these single-disease frameworks frequently results in complex, uncoordinated, and potentially hazardous pharmacological regimens, the combined risks of which have not been systematically evaluated [1,2].

The clinical consequences of polypharmacy extend far beyond the logistical complexity of managing intricate medication schedules in older adults. A robust and growing body of evidence consistently links it to elevated rates of adverse ADRs, clinically significant DDIs, iatrogenic falls, acute delirium, accelerated cognitive deterioration, suboptimal adherence, and avoidable emergency department (ED) presentation and hospitalization. A substantial and preventable proportion of acute geriatric hospital admissions are directly attributable to drug-related problems (DRPs), which can be meaningfully reduced by structured prescribing oversight, systematic medication reviews, and proactive deprescribing [3]. The resulting dual burden harms individual patients and escalates healthcare expenditures through prolonged hospital stays and redundant investigations, rendering polypharmacy not only a clinical problem but also a health system-level priority.

These risks are materially compounded by the physiological changes associated with normal and pathological aging. Declining renal and hepatic function alters the pharmacokinetic profile of many drugs, increasing the propensity for accumulation and toxicity, whereas pharmacodynamic alterations modify responses to specific drug classes, notably sedating and anticholinergic agents, which are independently associated with falls and cognitive impairment [4]. Such vulnerabilities demand a level of clinical scrutiny that transcends simple prescription counting; they necessitate a systematic, individualized evaluation of each agent’s active indication, safety profile, potential interactions, and alignment with the patient’s goals of care, functional status, and remaining life expectancy.

Despite the well-established risks, addressing polypharmacy in practice remains inherently difficult. Fragmented care pathways, severe time constraints during consultations, suboptimally integrated electronic health records (EHRs), and clinical inertia among prescribers present persistent structural challenges. Furthermore, patient anxiety regarding symptom recurrence or medication withdrawal, often shared by clinicians, frequently stalls the necessary medication reductions. Although validated frameworks such as the Screening Tool of Older People’s Prescriptions (STOPP) and Screening Tool to Alert to the Right Treatment (START) criteria provide evidence-based instruments for identifying potentially inappropriate medications (PIMs) and potential prescribing omissions (PPOs), their systematic and consistent application across diverse clinical settings remains inconsistent [5,6].

**Figure 1:**
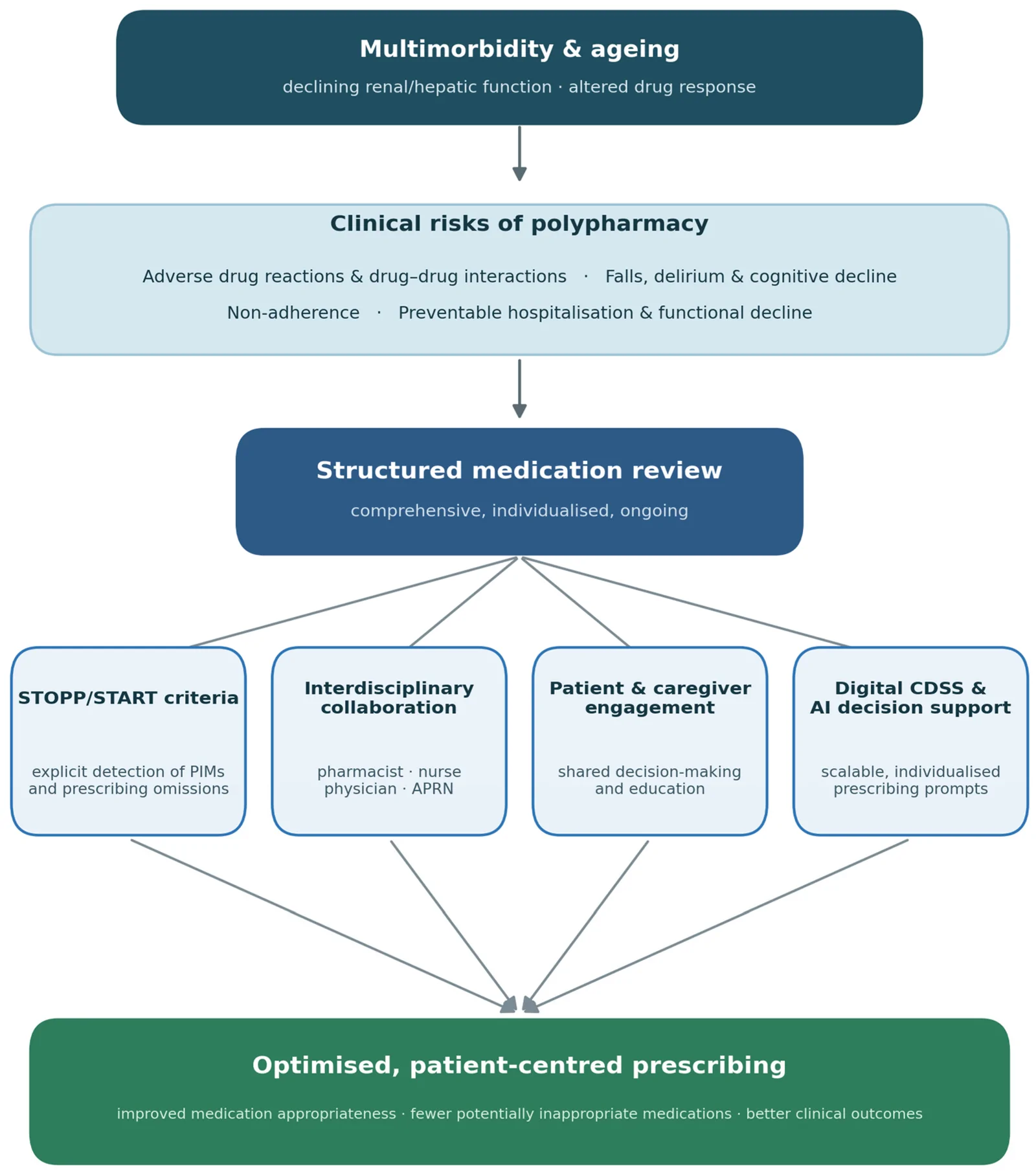
Conceptual framework for management of polypharmacy in older adults.

Against this background, this structured narrative literature review provides a comprehensive synthesis of empirical evidence on the epidemiology, clinical consequences, and management of polypharmacy in adults aged ≥65 years. It examines structured deprescribing algorithms, interdisciplinary co-management models, patient–caregiver educational frameworks, and digital health innovations, with explicit attention to the barriers and facilitators shaping real-world implementation. The review is guided by the premise that multicomponent, integrated interventions, combining structured clinical reviews, validated screening tools, interdisciplinary team-based workflows, and digital CDSSs, tend to achieve greater improvements in prescribing appropriateness and larger reductions in adverse outcomes than uncoordinated, discipline-specific care [7]. The secondary aim is to appraise the overall strength and consistency of the current evidence base and highlight persistent gaps, particularly regarding long-term patient-reported outcomes (PROMs) and cost-effectiveness data, to inform clinical practice across inpatient, primary care, home care, and transitional care settings for advanced practice registered nurses (APRNs), pharmacists, and prescribing clinicians, as summarized in the conceptual framework presented in Figure 1.

Multimorbidity and age-related physiological changes drive the clinical risk of polypharmacy. These issues are addressed through a structured medication review operationalized by four interacting components: the STOPP/START criteria, interdisciplinary collaboration, patient and caregiver engagement, and digital clinical decision support, converging on optimized patient-centered prescribing (Figure 1).

## 2. Methods

A structured narrative literature review was conducted to synthesize the current evidence on strategies for managing polypharmacy in elderly patients. This methodological approach was deliberately selected over a formal systematic review to permit a broader interpretive exploration of the conceptual, clinical, and contextual dimensions of the topic while maintaining transparency and rigor in the source selection, appraisal, and synthesis. The review was conducted and reported in accordance with the established principles of structured narrative literature reviews in clinical health research.

### 2.1. Study Design

This review was designed to identify, synthesize and discuss recent evidence on the incidence, risk profile, and clinical management of polypharmacy in adults aged ≥65 years. Consistent with the structured narrative literature review methodology, the synthesis integrated findings from diverse study designs, including randomized controlled trials (RCTs), cluster RCTs, study protocols, prospective and retrospective cohort studies, observational cross-sectional analyses, qualitative investigations, and mixed-methods evaluations, with a primary focus on deprescribing interventions, interdisciplinary care models, patient and caregiver education, and digital health technologies. A narrative synthesis approach was adopted given the heterogeneity of the included study designs, populations, and outcome measures, which precluded quantitative pooling.

### 2.2. Search Strategy

A comprehensive search strategy was developed and systematically applied across three major academic databases: PubMed, Scopus, and the Web of Science. Boolean logic was used to combine the Medical Subject Headings (MeSHs) with free text terms. Search strings were adapted to the indexing conventions and controlled vocabulary of each database to maximize the retrieval sensitivity and specificity. The overall search architecture addressed four thematic domains:

- Target population and core condition: (“polypharmacy” OR “hyper-polypharmacy” OR “inappropriate prescribing”) AND (“elderly” OR “older adults” OR “geriatric” OR “aged 65 and over”).
- Clinical outcomes and vulnerabilities: AND (“adverse drug reactions” OR “drug-drug interactions” OR “falls” OR “cognitive decline” OR “frailty”).
- Intervention frameworks: AND (“deprescribing” OR “medication review” OR “medication management” OR “STOPP/START criteria” OR “Medication Appropriateness Index”).
- Care models and technology: AND (“interdisciplinary collaboration” OR “pharmacist-led” OR “nurse-led” OR “clinical decision support systems” OR “CDSS”).

The strategy was structured around four thematic dimensions: pharmacological risks and outcomes (hospital admissions, falls, cognitive decline), deprescribing methods (gradual dose reduction, collaborative decision-making, digital tools), interdisciplinary approaches (pharmacists, nurses, primary care physicians), and patient- and caregiver-focused education and adherence monitoring.

The search was confined to primary, peer-reviewed research published between January 2016 and June 2026. All secondary research, including systematic reviews, meta-analyses, scoping reviews, and narrative opinion pieces, were excluded. Hand-searching of the reference lists of high-impact articles identified during the primary search was performed to ensure comprehensive capture of the data.

### 2.3. Eligibility Criteria

#### 2.3.1. Inclusion Criteria

Studies were eligible if they (1) enrolled adults aged ≥65 years with documented polypharmacy or who had undergone formal medication review; (2) applied validated prescribing review tools, including but not limited to STOPP/START criteria (versions 1 or 2), the Beers criteria, or the Medication Appropriateness Index (MAI); (3) reported medication review, deprescribing, or other structured medication-management approaches in clinical settings; (4) were peer-reviewed and published in English between January 2016 and June 2026; and (5) employed quantitative, qualitative, or mixed-methods designs that reported clinical, prescribing process, safety, or patient-centered outcomes.

#### 2.3.2. Exclusion Criteria

Studies were excluded if they (1) focused exclusively on patients aged <65 years; (2) did not address polypharmacy or structured deprescribing; (3) were editorials, opinion pieces, conference abstracts, or commentaries lacking full primary data; (4) constituted secondary research (systematic reviews, meta-analyses, scoping reviews) to maintain grounding in primary evidence; (5) were not published in English; (6) examined only a single isolated medication or therapeutic class rather than broader polypharmacy management; or (7) were animal, laboratory, or in vitro experimental models. Duplicate records identified during or after de-duplication were excluded.

### 2.4. Study Selection and Synthesis

Records retrieved from the three databases were screened by title and abstract against the eligibility criteria, and potentially relevant articles were then examined in full text before a final decision on inclusion. The reference lists of key articles were hand-searched to identify additional sources that were not retrieved by the electronic search. In total, 40 primary studies were included in the synthesis. Given the extensive body of literature on polypharmacy, this figure reflects a purposive rather than an exhaustive selection, prioritizing the most clinically relevant and contemporary primary evidence across the core thematic domains of polypharmacy management. Given the heterogeneity of study designs, populations, and outcome measures, the evidence was integrated narratively rather than pooled statistically, with studies grouped by thematic domain and, were helpful, by study design to aid interpretation. The objective was not exhaustive retrieval of evidence but the identification of the most clinically informative contemporary primary studies to support an interpretative narrative synthesis.

Data were extracted from each included study using a standardized extraction framework encompassing the following: study design and country, target population and sample size, intervention type and duration, data sources and measurement instruments, and principal findings pertaining to prescribing appropriateness, medication safety, or patient-centered outcomes. When multiple publications reported the same trial (e.g., a protocol and its results article), both were included and presented together to capture the design rationale alongside the outcome findings.

Data extraction was performed independently by two researchers using a predefined, standardized extraction form. Each reviewer extracted data from each included study without prior access to the other reviewers’ outputs. Discrepancies between the two extraction sets were identified through structured cross-comparison and resolved by consensus discussion; where consensus could not be reached, a third author acted as an independent reviewer. The extraction form captured (1) bibliographic identifiers and study design; (2) country of conduct and clinical setting; (3) sample size and participant characteristics (age, sex distribution, comorbidity profile, and medication burden); (4) intervention type and duration; (5) primary and secondary outcome measures and instruments; and (6) principal findings regarding prescribing appropriateness, medication safety or patient-centered outcomes. No participant data from the protocol papers were incorporated into the synthesis, and these pairs are presented together in the evidence tables with explicit notation to make this relationship transparent. This procedure ensured that no participant data were double counted and that each trial contributed only one set of outcome estimates to the narrative synthesis.

### 2.5. Quality Assessment

Given the heterogeneous nature of the included study designs, no formal methodological quality assessment or risk-of-bias appraisal using validated instruments was performed. This review was designed as a structured narrative synthesis rather than a systematic review with formal evidence grading. Methodological strengths and limitations of the included studies were considered during the narrative synthesis and are discussed where relevant within the thematic sections and acknowledged in Section 4.4.

### 2.6. Ethical Considerations

No institutional review board (IRB) ethical clearance was required for this review, as it relied exclusively on the secondary analysis of previously approved, peer-reviewed, and publicly available primary research publications. No individual patient medical records or identifiable personal data were accessed or processed in this study. Ethical compliance was maintained throughout the study through rigorous citation standards, accurate and faithful representation of the original findings, and scrupulous avoidance of selective data reporting.

## 3. Results

The 40 primary studies included in this review comprise a substantial and methodologically diverse body of evidence examining polypharmacy management across geriatric care settings: 13 RCTs, cluster RCTs, or trial protocols; 7 prospective or retrospective cohort studies; 7 observational cross-sectional studies; 6 descriptive, validation, or conceptual analyses; and 7 qualitative studies. Overall, the synthesis demonstrates consistent evidence that structured multicomponent interventions produce meaningful and reproducible improvements in prescription appropriateness and medication safety. The findings were organized across seven thematic domains, with supporting evidence tables (Table 1, Table 2, Table A1, Table A2, Table A3 and Table A4) grouped by study design to reduce data duplication.

### 3.1. Definition, Epidemiology, and Clinical Context of Polypharmacy

Clinical evidence confirms that a purely numerical definition of polypharmacy, the concurrent use of ≥5 medications, is insufficient for comprehensive geriatric risk assessment. A conceptually important and clinically actionable distinction has emerged in the literature between ‘appropriate polypharmacy,’ in which complex regimens are evidence-based, clinically optimized, and concordant with the patient’s goals of care, and ‘inappropriate polypharmacy,’ characterized by the accumulation of unnecessary, redundant, or potentially harmful medications whose cumulative risk outweighs the clinical benefit [8,9].

Two additional constructs require clinical attention. Hyper-polypharmacy, typically defined as the concurrent use of ≥10 medications, exponentially amplifies the risk of drug interactions and the compounding burdens of cognitive load, adherence failure and functional decline. In a nationwide South Korean cohort of 7,358,953 older adults, Cho et al. [1] found that hyper-polypharmacy (>5 drugs for >90 days) was prevalent in 11.9% of the population, and polypharmacy (≥5 drugs for ≥90 days) in 47.8%, with rates strongly associated with male sex, advancing age, and greater comorbidity burden. The ‘prescribing cascade, in which an unrecognized ADR is misdiagnosed as a new clinical condition, triggering an additional prescription and escalating iatrogenic risk, represents a distinct and underappreciated mechanism of avoidable harm that any structured review process must actively identify [8,9].

Epidemiological data reveal consistently high and rising rates of polypharmacy across international healthcare contexts, with considerable variation reflecting differences in primary care organization, pharmacist-led review capacity, national prescribing guidelines, and EHR infrastructure. Pharmacokinetic and pharmacodynamic changes associated with aging, principally declining renal and hepatic function and altered receptor sensitivity, further amplify drug exposure and harm risk, making routine, structured medication review a clinical imperative rather than an optional quality measure [4].

### 3.2. Clinical Risks and Consequences

The consequences of polypharmacy in older adults are numerous, serious, and well documented across diverse clinical settings. Foremost among these are ADRs and DDIs: as the number of concurrent medications increases, the probability of pharmacokinetic or pharmacodynamic interactions rises in a non-linear, exponential fashion, producing toxicity, reduced therapeutic efficacy, or both, outcomes that are particularly hazardous in a population with diminished physiological resilience [8,10]. Among 552 older cancer patients receiving chemotherapy, Oliveira et al. [10] identified polypharmacy in 88.4%, DDIs in 76.45%, and severe DDIs in 56.16%, illustrating the extraordinary interaction burden that can accumulate in clinically complex, multiply prescribed populations.

Sedating and anticholinergic agents are of particular concern because they are independently associated with falls, delirium, urinary retention, and accelerated cognitive decline. When used in combination, as commonly occurs in multimorbidity, these risks are substantially compounded. Consequently, the cumulative anticholinergic burden has emerged as a clinically important dimension of prescribing assessment, complementing simple drug counts and reinforcing the inadequacy of purely quantitative definitions [2,10].

Drug-related hospitalizations have major clinical and economic consequences. Lea et al. [3], in an observational study of 450 multimorbid inpatients in Norway, demonstrated that polypharmacy and inappropriate prescribing were the primary drivers of preventable drug-related hospital admissions, with estimates of preventable admissions ranging from approximately 10 to 30% across the literature. The ED context is particularly revealing in this regard. Salvi et al. [11], in a prospective cohort study of 2057 older ED patients, found polypharmacy in 30.3% and excessive polypharmacy in 17.8%, establishing ≥6 concurrent drugs as the optimal cut-off for predicting adverse outcomes, and demonstrating that polypharmacy independently predicted mortality, ED return, and hospital admission at six months. Polypharmacy is equally prevalent and challenging in palliative care settings. Granger et al. [12], analyzing PAL-HF trial data from 150 patients with advanced heart failure, found that polypharmacy was universal at baseline and increased in both the intervention and control arms, highlighting the profound difficulty of medication rationalization at the end of life.

Non-adherence represents a further, often underappreciated consequence of polypharmacy in older adults. Complex regimens involving multiple drugs, diverse dosing frequencies, and varied routes of administration challenge patient understanding and compliance, particularly among patients with limited health literacy or cognitive impairment. Chau et al. [13], in a prospective cohort of 6346 older adults across 29 Hong Kong residential care homes, found that PIM use (12-month prevalence 34.5%) was independently associated with hospitalization (OR 1.73, 95% CI 1.54–1.69), with the risk increasing significantly with a greater PIM burden (OR 2.17 for >1 PIM). These data underscore the clinically severe, economically significant, and critically preventable consequences of polypharmacy.

### 3.3. Barriers to Appropriate Prescribing

Efforts to optimize prescribing in older adults are confronted by barriers operating simultaneously at the patient, prescriber, and health system levels, a tripartite architecture that renders polypharmacy management inherently resistant to single-level interventions.

At the patient level, resistance to medication reduction is common and psychologically understandable: many older adults associate their medications with symptom control and stability and reasonably fear that discontinuation will precipitate relapse or deterioration. Limited health literacy and uncertainty regarding the purpose of individual agents compound this tendency, while the frequent and undisclosed use of herbal remedies and over-the-counter (OTC) products introduces interaction risks that prescribers cannot address without disclosure [14]. Qualitative research reinforces this picture: Zechmann et al. [6], in an interview study of 19 older, multimorbid Swiss patients, found that while none felt devalued by deprescribing discussions, conservatism and fragmented care coordination were identified as significant impediments, and patients strongly valued the continuity of care.

At the prescriber level, clinical inertia, the continuation of existing prescriptions in the absence of a compelling clinical reason to change, is a well-recognized impediment, reinforced by time constraints, the absence of structured review protocols, liability concerns regarding discontinuation, and limited training in geriatric pharmacology. Schmidt-Mende et al. [15], in a qualitative study nested within an RCT involving 194 GPs and 113 nurses in Swedish primary care, identified five overarching themes: the clinical complexity of managing multimorbid elderly patients, perceived conflicts between guideline recommendations and real-world practice, practical implementation constraints, multistep nature of medication review, and perceived threats to professional autonomy. These findings reveal that deprescribing is not merely a technical challenge but also a professional and relational one. The ED context is particularly problematic for this reason. Lee et al. [16], through focus groups and interviews with 28 ED clinicians and patients in the USA, identified time pressure, inadequate staffing, difficulty in obtaining complete medication histories, and the absence of structured deprescribing protocols as the principal barriers in this high-acuity, time-limited setting.

At the health system level, fragmented care pathways and the absence of seamlessly integrated EHRs create substantial risks for errors, particularly during care transitions. Mercer et al. [5], in a mixed-methods study across 28 healthcare sites in four Canadian provinces, found that pharmacists frequently lacked access to crucial information including drug indications and adherence histories—within existing EHR systems, severely impeding effective interdisciplinary collaboration and exposing patients to duplication and omission errors. Although CDSSs hold theoretical promise for bridging these information gaps, real-world implementation is commonly hampered by poor usability, inadequate workflow integration, insufficient clinician training, and alert overload fatigue [17].

### 3.4. Deprescribing and Structured Prescribing Tools

Deprescribing, the systematic, clinically supervised reduction, tapering, or discontinuation of medications that are no longer beneficial, whose risks outweigh their clinical value, or whose indications have become uncertain, is the cornerstone of polypharmacy management. Distinguished from unilateral patient discontinuation by its deliberate, evidence-guided nature, deprescribing incorporates a comprehensive medication review, shared decision-making, structured prioritization of discontinuation candidates, and ongoing monitoring for symptom recurrence or adverse effects [6]. Scott et al. [18] describe a validated five-step deprescribing process: (1) ascertain all current medications and the active indication for each agent; (2) assess the overall regimen for PIMs, PPOs, DDIs, and AD–s; (3) prioritize medications for discontinuation based on comprehensive risk-benefit analysis; (4) tailor decisions to individual patient values, preferences, and goals of care; and (5) monitor for symptom recurrence, withdrawal effects, and changes in adherence.

The STOPP and START criteria constitute the most widely validated explicit frameworks for geriatric medication review. STOPP identifies medications that are likely to cause harm in older adults, whereas START identifies clinically indicated medications that are commonly omitted from treatment regimens. Thevelin et al. [19] demonstrated that STOPP/START version 2 substantially outperformed version 1 in detecting clinically relevant PIMs and PPOs, increasing detection from 23% (v1) to 40% (v2), reinforcing the clinical imperative to use the most current validated iteration. Multiple large-scale trials have confirmed the practical value of these criteria. Within the OPERAM cluster RCT [7] (*n* = 2008), STOPP/START-guided interdisciplinary medication reviews embedded in structured ward discussions improved prescribing appropriateness in 35–40% of intervention patients and were associated with fewer drug-related hospitalizations over 12 months, although the reduction in overall drug-related admissions did not reach statistical significance at the primary endpoint. The OPTICA cluster RCT [20] (*n* = 323) similarly reported statistically significant improvements in medication appropriateness and clinically relevant PIM reduction, although the effects on the MAI and AOU at 12 months were inconclusive.

A critical, often underappreciated finding from RCT evidence is that improved prescribing process outcomes do not automatically translate into measurable patient-centered benefits, a process–outcome gap that represents a central and unresolved challenge for the field. Kaminaga et al. [21] demonstrated a further unintended consequence of in-hospital deprescribing: the proportion of patients with any PPO increased significantly from 52.9% to 77.7% following in-hospital medication reduction (*p* < 0.001), with older age as an independent risk factor (OR = 1.08). This finding compellingly illustrates that deprescribing focus must be balanced with simultaneous attention to underprescribing—a bidirectional challenge that the STOPP/START framework, with its explicit START component, is uniquely positioned to address. In the ReMInDAR trial [22], which integrated frailty assessment into pharmacist-led reviews in residential aged-care facilities, 37% of intervention participants achieved improved frailty scores, providing direct empirical evidence that medication optimization aligned with functional status yields clinically meaningful patient-level gains.

### 3.5. Interdisciplinary Collaboration in Medication Management

Polypharmacy cannot be effectively or safely managed by a single clinician acting in isolation. It demands a genuinely collaborative approach in which pharmacists, nurses, physicians, and APRNs contribute complementary and essential expertise: pharmacological knowledge, longitudinal therapeutic relationships, diagnostic authority, and frontline monitoring capacity, respectively. Evidence for pharmacist-led and team-based interventions was the most consistent and compelling in this review.

The OPERAM cluster RCT [7] provided the most rigorous large-scale evidence for interdisciplinary medication review, embedding STOPP/START-guided pharmacist–physician assessments within structured interdisciplinary discussions across four European healthcare systems. The intervention produced significant improvements in prescribing appropriateness, with 35–40% of patients achieving potentially inappropriate medication (PIM) reduction and a measurable reduction in drug-related hospitalizations within 12 months. Complementary evidence from a Swiss tertiary hospital retrospective cohort study by Studer et al. [23], which analyzed 4545 patients across 6072 hospital stays, demonstrated that pharmacist-led medication reconciliation combined with interdisciplinary ward rounds substantially reduced DRPs at discharge, a particularly critical and vulnerable transition point in care. In contrast, reconciliation alone produced only a non-significant trend toward benefit, powerfully underscoring the additive, non-substitutable value of structured interdisciplinary communication.

In a Taiwanese RCT, Lin et al. [24] evaluated pharmacist–physician collaborative medication therapy management (MTM) in 132 elderly patients with polypharmacy and demonstrated a 45.0% resolution of DRPs in the MTM group compared with 12.8% in the control group, alongside significantly lower healthcare costs and fewer hospital admissions, providing rare and valuable evidence linking pharmacist collaboration directly to cost reduction and patient outcomes. Qualitative inquiry by Rezahi et al. [25] confirmed the breadth and impact of pharmacist contributions within Ontario Family Health Teams, encompassing medication management (70.2% of roles), patient counseling and education (15.5%), and management of diabetes (39%), cardiovascular disease (22%), and chronic pain (17%).

Nurses, particularly APRNs, serve as critical frontline agents because of their sustained proximity to patients across inpatient, community, and transitional care settings. Steinman et al. [26], in a non-randomized controlled trial of 1218 high-risk older adults across 13 US/Israeli primary care clinics, demonstrated that a nurse-led educational and follow-up intervention (the Guided Care model) produced significantly more medication changes in the intervention group (adjusted difference 0.55, *p* = 0.001) and greater attention to symptomatic medications without increasing the total drug burden, providing evidence that nursing engagement improves regimen quality rather than merely reducing medication counts. Sun et al. [27] demonstrated, in a pilot pre–post intervention with 45 home care nurses in Ontario, that targeted deprescribing education significantly improved nursing pharmacology knowledge, self-efficacy, and readiness to initiate deprescribing discussions. Little et al. [28], in a qualitative study across three US long-term care facilities, identified three foundational themes for sustainable nurse-led deprescribing—building therapeutic trust, identifying patient-specific motivating factors, and standardizing institutional supportive processes—generating six actionable clinical steps for implementation in this setting.

### 3.6. Patient Education, Adherence, and Shared Decision-Making

Patient education, when delivered as a targeted and individualized component of a broader medication management strategy, is an effective and modifiable lever for improving adherence and reducing DRPs in the elderly population. A community pharmacist-led RCT by Messerli et al. [29], conducted across 54 Swiss pharmacies with 450 patients on polypharmacy, found that the Polymedication Check improved subjective adherence (*p* = 0.028) and successfully resolved approximately one-third of active DRPs, although it did not produce a statistically significant improvement in objective adherence measured by the Medication Possession Ratio (MPR). These nuanced findings caution against equating medication reviews with automatic adherence improvement and highlight the critical importance of selecting outcome measures that reflect the intended intervention mechanisms (Table 1).

**Table 1:** Intervention Studies: RCTs, Cluster RCTs, RCT Protocols, and Non-Randomized Intervention Studies (*n* = 13).

Study (Year) & Country	Design, Setting & Follow-Up Duration	Population & Sample (*N*)	Intervention Description	Key Findings (Primary & Outcomes)
Messerli et al. (2016) [29] Switzerland	Prospective parallel RCT 54 community pharmacies, Switzerland Duration: 28 weeks	Patients using ≥4 prescribed medicines for >3 months *N* = 450 (218 intervention; 232 control)	Structured pharmacist-led Polymedication Check comprising two pharmacist visits combined with face-to-face adherence counseling	Fewer inappropriate medications and improved adherence; one-third of drug-related problems resolved at 6 months
Lin et al. (2018) Taiwan [24]	Prospective parallel RCT Primary care/hospital, Taiwan Duration: 6 months	Geriatric outpatients (≥65 years) on ≥5 medications *N* = 132	Coordinated pharmacist–physician MTM: collaborative medication review, DRP identification, and shared decision-making to optimize regimens	The MTM cohort resolved 45.0% of DRPs, with significantly lower healthcare costs and fewer hospital readmissions in the intervention arm.
Steinman et al. (2018) [26] USA/Israel	Non-randomized controlled clinical trial (clinic-level allocation) 13 primary care clinics (7 intervention, 6 control) Duration: 9 months	Vulnerable older adults with ≥3 chronic conditions *N* = 1218	Nurse-led medication review, monitoring, and systematic telephone follow-up (Guided Care model); clinic-level allocation	The intervention group showed significantly more medication changes, including more appropriate changes to symptomatic medications, without increasing the total drug count.
Blum et al. (2021) [7] OPERAM Multinational	Multinational cluster RCT (110 clusters; 4 European countries) Hospital inpatient units Duration: 12-month	Hospitalized adults ≥ 70 years with multimorbidity and polypharmacy *N* = 2008 (1102 intervention; 906 control)	STOPP/START-guided interdisciplinary medication review (STRIP methodology) using the electronic STRIP-Assistant CDSS; physician–pharmacist collaborative review teams	Prescribing appropriateness improved in 35–40% of intervention patients (significant PIM reductions), fewer drug-related hospital admissions over 12 months, and no statistically significant reduction in overall drug-related admissions at the primary endpoint was observed.
Lim et al. (2020) [22] ReMInDAR Australia	RCT protocol 39 residential aged-care facilities, Australia Duration: 12 months (pharmacist visits every 8 weeks)	Aged-care facility residents with polypharmacy (≥5 medications) *N* = 248 planned across 39 facilities	Pharmacist-led, frailty-informed medication review conducted every 8 weeks; sessional pharmacist monitoring focused on frailty index optimization	Protocol target: Reducing frailty index through pharmacist monitoring. Reported outcomes: 37% of the intervention participants achieved improved clinical frailty scores, and fewer adverse drug reactions were documented relative to the control group.
McDonald et al. (2022) [30] MedSafer Canada	Multi-center cluster RCT 11 acute care hospitals, Canada Duration: Hospital stay + 30-day ADE	Hospitalized adults ≥ 65 years on ≥5 medications *N* = 5698 (2827 intervention; 2871 control)	Electronic decision support system (MedSafer) generating personalized, patient-specific deprescribing opportunity reports at hospital discharge for the treating medical team	No statistically significant reduction in 30-day adverse drug events was observed, indicating a process–outcome gap that requires further investigation to determine the cause.
Jungo et al. (2023) [20] OPTICA Switzerland	Cluster RCT (43 GP clusters) Primary care practices, Switzerland Duration: 12-month	Multimorbid primary care patients (≥65 years) on ≥5 medications *N* = 323 (163 intervention; 160 control)	GP-led structured medication assessment with pharmacist support using eCDSS (STRIPA/STOPP-START), integrated with shared decision-making	There was a statistically significant improvement in medication appropriateness, clinically relevant PIM reduction, ~1 deprescribing recommendation implemented per patient, and the effect on MAI/AOU scores at 12 months remained inconclusive.
Studer et al. (2023) [23] Switzerland	Retrospective intervention cohort Internal medicine wards, regional hospital, Switzerland Duration: Hospital stay (2016–2019 routine data)	Consecutive internal medicine inpatients *N* = 4545 patients (6072 hospital stays)	Pharmacist-led medication reconciliation at admission, combined with active participation in interdisciplinary ward rounds; four-group retrospective analysis	The combined strategy significantly reduced DRPs at discharge, whereas reconciliation alone showed only a non-significant trend toward benefit.
David et al. (2024) [31] USA	Parallel-group clinical RCT Primary care clinics, USA Duration: 3 months	36 PCPs (16 intervention; 20 control) and 540 polypharmacy patients (126 intervention; 207 control)	Chronic Disease Management Test (CDMT): combined electronic testing of non-adherence and DDIs, generating actionable clinical alerts for primary care physicians	Intervention PCPs: significantly higher detection rates for non-adherence; high-risk DDIs corrected in 28% of cases.
Prados-Torres et al. (2017) [32] Multi-PAP Spain	Cluster RCT protocol Primary care centers, Spain Duration: 6- and 12-month outcomes	Multimorbid primary care patients ≥ 65 years on ≥5 drugs *N* ≈ 400 planned (200 per arm);	Family physician (FP) training in Ariadne principles combined with structured FP–patient interviews targeting medication appropriateness; cluster-randomized by FP	42% achieved improved prescribing appropriateness; fewer omissions
Jungo et al. (2019) [33] OPTICA Protocol Switzerland	Cluster RCT protocol Primary care, Switzerland Duration: 9–12 months	Multimorbid primary care patients ≥ 65 years on ≥5 regular medications 42 GP clusters planned	OPTICA structured medication assessment protocol: GP cluster randomization integrating a pharmacist-supported eCDSS (STRIPA/STOPP-START) into routine primary care workflows	The operational protocols, CDSS algorithms, and clinical endpoints were formally established and subsequently implemented by Jungo et al. [20].
Adam et al. (2019) [34] OPERAM Protocol Multinational	Cluster RCT protocol Hospitals in 4 European countries Duration: Index admission + 12-month	Hospitalized adults ≥ 70 years with multimorbidity and polypharmacy *N* = 2008 planned	STOPP/START-guided interdisciplinary medication review via STRIP methodology and a STRIP-Assistant CDSS; cluster-randomized by attending physician; primary endpoint: drug-related readmission at 12 months	The operational framework, blinding strategies, randomization procedures, and adjudication methods were implemented by Blum et al. [7].
Sun et al. (2021) [27] Canada	Pre–post quantitative intervention pilot study Homecare setting, Southern Ontario, Canada Duration: Single-session training evaluation	Homecare registered nurses working with older community-dwelling adults *N* = 45	Focused educational modules on geriatric pharmacology, evidence-based deprescribing guidelines, and structured communication strategies for medication reduction conversations	Training significantly improved pharmacology knowledge, self-efficacy scores, and readiness to initiate a clinical deprescribing review. The program was deemed suitable for broader-scale implementation.

David et al. [31], in an RCT of 540 patients across 36 US primary care practices, evaluated a Chronic Disease Management Test (CDMT) combining electronic non-adherence and DDI testing. Intervention physicians demonstrated substantially higher detection and action rates for non-adherence (69.1% vs. 20.3%, *p* < 0.001) and DDIs, with high-risk DDIs corrected in 28% of cases, illustrating that equipping clinicians with structured detection tools and actionable data meaningfully and measurably changes prescribing behavior. Herbal remedies and OTC products represent a frequently under-recognized and clinically significant risk dimension; their undisclosed use creates pharmacokinetic and pharmacodynamic interactions that are invisible to prescribers, often because patients do not consider these substances medications. Comprehensive counseling programs that systematically review all substances consumed were associated with the identification and reduction in drug–herb interaction risk in the included studies [26,29].

Shared decision-making, in which medication changes are anchored in the patient’s fully informed preferences, values, and quality-of-life goals rather than unilaterally imposed by clinicians, is both ethically appropriate and a well-supported determinant of improved adherence and treatment outcomes in complex older populations. Patients who actively participate in medication decisions demonstrate better adherence, fewer regret-related discontinuations, and greater engagement in follow-up monitoring. Engaging caregivers and proxies extends these benefits to patients with cognitive impairment or limited capacity, which is a particularly important consideration in residential and long-term care contexts [25,28]. Clear, simplified communication and deliberate caregiver involvement are especially valuable during high-risk transitions, such as hospital discharge, when regimens frequently change, and patients are most vulnerable to errors [5,35].

### 3.7. Digital Health and Clinical Decision Support

Digital health technologies, and CDSSs in particular, have emerged as promising mechanisms for improving the reliability, scalability, and reach of deprescribing, addressing the cognitive limitations and information processing bottlenecks inherent in managing complex polypharmacy at scale. The MedSafer study [30], a large cluster RCT across 11 Canadian hospitals with 5698 hospitalized older adults, provided the strongest evidence in this domain: patients receiving computer-generated individualized deprescribing opportunity reports at hospital discharge had significantly higher rates of discontinuation of at least one PIM compared with those receiving usual care. However, this intervention did not achieve a statistically significant reduction in 30-day adverse drug events, starkly illustrating the process–outcome gap between medication discontinuation rates and downstream patient-centered harm reduction, and underscoring the need for adequately powered, longer follow-up trials with patient-relevant primary endpoints.

The OPTICA cluster RCT [20] integrated an eCDSS (STRIPA/STOPP-START) into structured GP–pharmacist medication assessments in Swiss primary care, and the CDMT trial [31] combined electronic DDI monitoring with personalized clinical feedback in primary care. Together, these studies affirm that digital tools function most effectively as structured clinical prompts embedded within established interdisciplinary review workflows, augmenting rather than substituting clinical judgment. The observational MULTIPAP study [14] demonstrated that even a relatively simple computer-assisted prescription alert system detected DDIs in 27% of 480 primary care patients and prompted prescription modifications in 19%, illustrating that technologically modest interventions can achieve clinically meaningful scale effects when appropriately integrated into the workflow.

Conceptual analyses by Băjenaru et al. [36] and Sopruchi et al. [37] provide a forward-looking context for more sophisticated AI-driven CDSSs, identifying the potential for individualized, data-driven recommendations. These analyses also identified significant barriers to responsible clinical deployment—data quality limitations, algorithmic bias, lack of decision transparency, and low clinician trust—that must be systematically addressed before AI-driven tools can be safely integrated into geriatric prescribing practice. Zhai et al. [17], applying the FITT (Fit between Individuals, Task, and Technology) framework, conducted 200 h of participatory observation and 21 semi-structured interviews in a 2000-bed Shanghai hospital and identified 12 categories of barriers and facilitators to nursing CDSS adoption. Their most important finding—that administrative leadership and institutional commitment were the primary determinants of sustainable implementation—converges with the broader implementation science literature in emphasizing that governance, not technology quality alone, determines whether digital tools are sustainably adopted in clinical practice.

**Table 2:** Studies Applying STOPP/START Criteria: Clinical Findings (6 studies).

Study (Year)	STOPP Applied	START Applied	Key Finding
Thevelin et al. (2019) [19]	✓	✓	STOPP/START version 2 increased clinical detection of PIMs and PPOs from 23% (v1) to 40% (v2); updated criteria substantially improved identification of clinically relevant drug-related errors
Sallevelt et al. (2022) [38]	✓	✓	8.7% of 963 OPERAM patients readmitted with a potentially preventable DRA; single in-hospital review failed to detect 48% of medication errors present at readmission; of detectable errors, ~25% received no recommendation and ~25% of recommendations were not implemented
Abukhalil et al. (2022) [39]	✓	✓	66.8% of hospitalized elderly (≥65 years) had potentially inappropriate prescriptions; polypharmacy present in 44.5%; STOPP/START demonstrated high detection utility in a resource-limited context
Kaminaga et al. (2021) [21]	✓	✓	In-hospital deprescribing increased PPOs from 52.9% to 77.7% (*p* < 0.001); advanced age was an independent risk factor (OR 1.08); the 2015 STOPP/START criteria were applied; and the findings highlighted the bidirectional risk of unbalanced deprescribing.
Blum et al. (2021)—OPERAM [7]	✓	✓	STOPP/START-guided interdisciplinary review improved prescribing appropriateness in 35–40% of intervention patients; fewer drug-related hospital admissions over 12 months; STRIP-Assistant CDSS facilitated structured real-time application
Jungo et al. (2023)—OPTICA [20]	✓	✓	eCDSS incorporating STOPP-START algorithms improved prescribing appropriateness; ~1 deprescribing recommendation implemented per patient; effect on MAI/AOU scores at 12 months remained inconclusive despite process improvements

Note. STOPP, Screening Tool of Older People’s Prescriptions; START, Screening Tool to Alert to the Right Treatment; PIM, potentially inappropriate medication; PPO, potential prescribing omission; DRA, drug-related admission; CDSS, clinical decision support system. The studies were arranged chronologically.

## 4. Discussion

Across the 40 included primary studies, a convergent and clinically important pattern emerged: structured multicomponent interventions that combined validated prescribing review tools, pharmacist expertise, sustained interdisciplinary collaboration, patient education, and digital decision support produced the most robust and reproducible improvements in prescribing appropriateness and medication safety in older adult populations. This synthesis provides strong and consistent evidence that polypharmacy in older adults is clinically manageable when addressed using coordinated, systematic, and patient-centered strategies. The findings both corroborate and substantially extend the established consensus in geriatric pharmacology by documenting the specific disaggregated contribution of each management component across 40 contemporary primary studies.

### 4.1. Efficacy of Pharmacist-Led Interventions and Validated Prescribing Tools

The evidence affirms, with substantial consistency, that pharmacist-led medication reviews, whether delivered as a standalone community service or embedded within interdisciplinary inpatient care, are the most reliably effective strategy for reducing polypharmacy-related harm. The landmark OPERAM cluster RCT [7] and OPTICA cluster RCT [20] together provide large-scale, methodologically rigorous evidence that pharmacist involvement within interdisciplinary teams yields measurable and reproducible gains in prescribing appropriateness and PIM reduction across diverse European healthcare systems. The depth of pharmacological expertise, familiarity with evidence-based prescribing guidelines, and capacity to identify interaction risks position pharmacists as non-substitutable members of the geriatric care team at every clinical interface.

The STOPP/START criteria demonstrate sustained and evolving utility as structured assessment frameworks, with version 2 offering markedly superior sensitivity for clinically relevant PIMs and PPOs compared with version 1 [19]. Their integration into clinical workflows, whether at pre-admission review, structured ward rounds, or primary care consultations, is consistently associated with improved prescription quality. However, a critical and persistently underemphasized finding across the included studies is that improved prescribing process outcomes do not automatically translate into measurable, patient-centered, clinical benefits. Neither MedSafer [30] nor OPERAM [7] achieved statistically significant reductions in adverse drug events or drug-related admissions at their primary endpoints, despite demonstrating unambiguous improvements in prescription appropriateness. This process–outcome gap represents the most important and underexplored challenge in geriatric polypharmacy research and compellingly underscores the need for adequately powered, long-term trials using patient-centered primary endpoints, including hard clinical outcomes, patient-reported quality of life, and functional independence.

Patient education and adherence support are modifiable, relatively low-cost strategies that complement structured medication reviews by reliably amplifying their benefits when delivered in a personalized, sustained, and health literacy-sensitive manner. Nurse- and pharmacist-led education, combined with deliberate caregiver involvement, reduces errors, improves treatment adherence, and fosters active patient participation in medication decisions—factors that collectively sustain prescribing improvements beyond the clinical encounter itself [26,28].

### 4.2. The Role of Technology and Systemic Barriers

Digital health technologies, particularly CDSsS, offer a uniquely scalable mechanism for extending the reach, reliability, and consistency of deprescribing. The MedSafer cluster RCT [30] established strong proof-of-concept evidence that computer-generated deprescribing recommendations can significantly increase the rate of appropriate PIM discontinuation at hospital discharge. AI-powered systems extend this potential by generating individualized, data-driven recommendations that dynamically account for the patient’s full clinical, functional, and pharmacological profile in ways that static, rule-based algorithms cannot achieve. However, realizing this potential in practice depends on resolving foundational barriers: data quality and completeness, algorithmic bias and equity, interpretability and transparency of decision logic, sustained clinician trust, and robust governance frameworks—conditions that are not yet consistently met in current AI implementations [36,37].

System-level barriers, principally fragmented care pathways and the absence of comprehensively integrated EHRs, remain major under-addressed structural impediments to medication safety in elderly populations. Poor information continuity during care transitions is a well-established driver of prescription errors, particularly at the hospital–community interface (HCI). Structured medication reconciliation, pharmacist-led ward round participation, and targeted discharge counseling substantially reduce DRP rates at this high-risk juncture [5,23]. Critically, CDSS implementation must be treated as an organizational change process requiring co-design with end users, iterative usability testing, targeted staff training, phased workflow integration, and sustained institutional commitment—investments that are prerequisites for sustainable adoption, not optional enhancements. The FITT framework analysis by Zhai et al. [17] powerfully illustrates that the determinants of successful digital tool adoption are primarily organizational and cultural rather than technical issues.

### 4.3. Comparison with Existing Literature and Identified Gaps

A fundamental limitation of the current literature is its reliance on rigid chronological age thresholds (≥65 or ≥70 years), which fail to capture the profound heterogeneity of the older adult population with respect to frailty status, functional capacity, cognitive reserve, care goals, and life expectancy. These clinically critical dimensions are inadequately represented in most study designs, eligibility criteria, and clinical guidelines. The ReMInDAR trial protocol [22], with its explicit integration of frailty assessment into pharmacist-led medication review decisions, represents a methodologically important and clinically logical exception that warrants replication and wider adoption. Future trial designs should employ individualized, frailty-stratified eligibility criteria and outcome measures rather than applying uniform deprescribing targets to an inherently and substantially diverse patient population.

Several persistent evidence gaps substantially limit the evidence base and should inform future research priorities. First, reliable large-scale cost-effectiveness data are sparse. Although the Taiwanese MTM trial [24] provides encouraging preliminary evidence of health–economic benefits alongside clinical improvement, economic evaluations of sufficient scale and methodological rigor to inform national funding and scaling decisions are absent from the literature, which is a significant constraint on the translation of health policy. Second, the predominance of intermediate process endpoints (PIM rates, medication counts, or drug-related admission rates) with relatively short follow-up periods (typically ≤12 months) provides limited insight into the outcomes that matter most to patients, such as functional independence, symptom burden, health-related quality of life, treatment burden and survival. Validated PROMs should be incorporated as primary or co-primary endpoints in future studies. Third, the near-exclusive representation of high-income, Western health systems limits the generalizability of current evidence; lower-resource settings face distinct structural, financial, and workforce challenges that necessitate context-specific research and specifically adapted implementation frameworks.

The fourth critical gap concerns the unintended consequences of deprescription. As demonstrated by Kaminaga et al. [21], in-hospital medication reduction can inadvertently and significantly increase PPOs when review protocols focus narrowly on eliminating PIMs without simultaneously and systematically reviewing clinically indicated omissions. Therefore, a balanced bidirectional review using tools that comprehensively address both the STOPP and START criteria is essential. Finally, the evidence base for APRN-led polypharmacy management, despite APRNs’ critical and irreplaceable frontline position, remains comparatively limited and warrants dedicated, adequately powered investigations.

### 4.4. The Strengths and Limitations of This Review

This review synthesizes a substantial and contemporary body of primary evidence (2016–2026) from multiple countries, clinical settings, and study designs, integrating conceptual, clinical, and implementation perspectives within a unified analytical framework. Its transparent search approach and structured tabular presentation of the key studies allow readers to trace the basis of each conclusion and calibrate the strength of the evidence for themselves. The explicit seven-domain thematic structure, combined with non-duplicated standardized evidence tables, facilitates clinical utility. This review also benefited from the methodological expertise of the senior researcher in evidence-based practice and geriatric pharmacotherapy.

The limitations of this review are due to its methodological design. As this review was conducted as a structured narrative synthesis, and because of the substantial heterogeneity of the included study designs, no formal methodological quality assessment or risk-of-bias appraisal using validated instruments was performed. Consequently, the findings should be interpreted in the context of a narrative synthesis rather than a formally graded body of evidence. The restriction to English-language publications may have excluded relevant research conducted and disseminated in other languages, potentially introducing a language publication bias. The exclusion of secondary literature, while deliberate and methodologically justified, may have omitted useful contextual synthesis. The considerable heterogeneity of populations, interventions, outcome measures, and follow-up periods across the 40 included studies precluded the quantitative pooling of effect estimates, and the interpretive nature of the narrative review means that the synthesis reflects the informed judgment of the reviewing team rather than statistical weighting. These considerations should be borne in mind when translating the findings into specific clinical practice or policy decisions.

An additional limitation is that the standardized extraction form, although systematically designed, was not formally pilot-tested prior to its deployment. This introduces the risk of inconsistent field interpretation, particularly given the methodological heterogeneity of the 40 studies included. Future structured narrative reviews on this topic should conduct a formal pilot phase on a representative subset of studies before full-scale data extraction.

### 4.5. Future Directions

Several developments are likely to reshape polypharmacy management in the coming decade. The most immediate is the maturation of artificial intelligence (AI)-supported deprescribing systems. Where current rule-based systems flag predefined drug–drug interactions or inappropriate medications, machine learning models trained on large, longitudinal prescribing datasets could move toward genuinely personalized recommendations by estimating an individual’s risk of a specific adverse event, ranking candidate medications for withdrawal, and anticipating the likely consequences of stopping or continuing a given drug. Realizing this potential responsibly will depend less on algorithmic sophistication than on the quality and representativeness of the underlying data, the transparency of the decision logic, and the degree to which clinicians trust and are willing to act on the output of the algorithm. Therefore, future work should pair model development with pragmatic implementation studies that examine how AI-generated recommendations are received and used at the point of care, rather than reporting predictive accuracy in isolation.

The second major priority is the transition from age-based to frailty-based prescriptions. Chronological age remains a poor proxy for physiological reserve, and the heterogeneity captured by frailty, functional status, cognition, and remaining life expectancy is far more relevant to whether a given regimen is beneficial or harmful. Embedding validated frailty measures directly into medication review workflows, as the ReMInDAR model began to do, would allow deprescribing decisions to be tailored to the individual rather than applied uniformly across an age band. Linked to this is the broader goal of personalized medication management, in which prescribing is continuously revisited against a patient’s changing goals of care and in which patient-reported outcomes are tracked as core endpoints rather than afterthoughts. Ultimately, the value of digital decision support will be determined by how well these tools are integrated into interoperable electronic health records and into the everyday routines of interdisciplinary teams. Therefore, the most promising future is one in which AI-supported tools, frailty-informed criteria, and shared decision-making operate as a single, coordinated system rather than as separate innovations.

## 5. Conclusions

Polypharmacy in older adults is a growing healthcare challenge driven by longer life expectancy and multiple chronic conditions. Polypharmacy is strongly linked to adverse drug reactions, harmful drug interactions, hospitalizations, functional decline, and reduced quality of life, many of which are preventable. Pharmacist-led medication reviews using tools such as STOPP/START improve prescribing quality, while collaboration among healthcare professionals and informed patients leads to better outcomes. Patient education tailored to individual needs helps to sustain improvements. Digital tools, such as clinical decision support systems and AI, can further enhance safe medication use; however, they require proper implementation and integration into existing systems.

Long-term progress depends on coordinated efforts across healthcare systems, including better electronic health records, improved care transitions, enhanced training in geriatric pharmacology, and personalized care models for older adults. Future research should focus on patient-centered outcomes, cost-effectiveness, and scalable technology-supported strategies for the management of complex cases.

Ultimately, the future of safe and effective polypharmacy management lies in frailty-based medication review, which tailors prescribing decisions to each patient’s functional status, goals of care, and life expectancy rather than chronological age or medication counts alone. Within this model, artificial intelligence and digital decision-support tools should support, but never replace, clinical decision-making by serving as structured prompts that inform, rather than dictate, prescribing choices. Above all, sustained interdisciplinary collaboration among pharmacists, nurses, physicians, and advanced practice registered nurses will remain the cornerstone of safe and effective polypharmacy management, ensuring that the complex needs of older adults are met through coordinated, patient-centered care and clinically vigilant care.

## Data Availability

All data analyzed are contained within publicly available peer-reviewed sources cited in the reference list.

## Abbreviations

The following abbreviations are used in this manuscript:

ADR	Adverse drug reaction
AI	Artificial intelligence
APRN	Advanced practice registered nurse
CDSS	Clinical decision support system
CKD	Chronic kidney disease
DDI	Drug–drug interaction
DRP	Drug-related problem
ED	Emergency department
EHR	Electronic health record
FITT	Fit between Individuals, Task, and Technology (framework)
OPERAM	Optimizing Therapy to Prevent Avoidable Hospital Admissions in Multimorbid Older Adults
OPTICA	Optimizing Pharmacotherapy in the Multimorbid Elderly in Primary Care
PIM	Potentially inappropriate medication
PPO	Potential prescribing omission
PROM	Patient-reported outcome measure
RCT	Randomized controlled trial
ReMInDAR	Reducing Medicine-Induced Deterioration and Adverse Reactions
START	Screening Tool to Alert to the Right Treatment
STOPP	Screening Tool of Older People’s Prescriptions

## Appendix A

**Table A1:** Longitudinal Cohort Studies (*n* = 7).

Study (Year) & Country/Study Aim	Design, Setting & Follow-Up Duration	Population & Sample (*N*)	Sampling Method & Data Source/Measurement	Key Findings (Primary & Outcomes)
Salvi et al. (2017) Italy [11] Aim: To verify polypharmacy as an independent risk factor for adverse post-ED outcomes in older adults.	Prospective observational cohort Emergency department, Italy Duration: 6-month follow-up	Older ED patients ≥ 65 years *N* = 2057 consecutive attendees	Consecutive enrolment; prescription histories from the preceding 3 months paired with prospective post-ED clinical event tracking (mortality, ED return, hospital admission)	Polypharmacy (6–9 drugs) was observed in 30.3% of patients, and hyper-polypharmacy (≥10 drugs) in 17.8% of patients. A cut-off of ≥6 drugs best predicted 6-month mortality and hospital admission. Polypharmacy was independently associated with adverse composite outcomes after multivariate adjustment.
Min et al. (2021) South Korea [4] Aim: To analyze the association between polypharmacy and renal hazards in pre-dialysis patients with chronic kidney disease (CKD).	Prospective multi-center cohort (KNOW-CKD registry) CKD specialty clinics, South Korea Duration: Mean follow-up 3.6 years	Pre-dialysis CKD patients (stages 1–5) *N* = 1913	Multi-center prospective KNOW-CKD clinical registry; longitudinal data on renal function markers, medication counts, and comorbidity burden	The prevalence of polypharmacy was 27.1%. Increased medication count was associated with a higher renal hazard (HR 1.056); however, the effect was fully mediated by the overall comorbidity burden and baseline kidney function, and no independent direct renal pharmacological hazard was identified.
Kaminaga et al. (2021) Japan [21] Aim: To investigate the unintended effects of in-hospital deprescribing on potential prescribing omissions (PPOs) in elderly inpatients.	Retrospective observational cohort Internal medicine ward, Japan Duration: Admission to discharge	Geriatric inpatients ≥ 65 years with polypharmacy (≥5 medications) *N* = 121	Consecutive enrolment; structured medical records review applying 2015 STOPP/START criteria at both admission and discharge	In-hospital deprescribing unintentionally increased the proportion of patients with any PPO from 52.9% to 77.7% (*p* < 0.001). Advanced age was an independent risk factor for PPOs (OR 1.08), highlighting a clinically important bidirectional prescribing trade-off.
Granger et al. (2022) USA [12] Aim: To describe polypharmacy patterns and evaluate the impact of a palliative care intervention on the medication burden in patients with advanced heart failure.	Observational cohort (secondary analysis of PAL-HF RCT dataset) Single academic center, USA Duration: Up to 24 weeks	Patients with advanced heart failure *N* = 150	Serial medication reviews extracted from PAL-HF RCT clinical trial records	Polypharmacy was universal at baseline (100%), and medication counts continued to rise in both arms over 24 weeks, regardless of palliative intervention, underscoring the difficulty of rationalization in end-stage diseases.
Doumat et al. (2023) Lebanon [2] Aim: To examine the association between polypharmacy and healthcare service utilization among community-dwelling older adults with comorbidities.	Retrospective database cohort Primary care, AUBMC, Lebanon Duration: Cross-sectional baseline with retrospective utilization tracking	Community-dwelling older adults ≥ 65 years with chronic comorbidities *N* = 496	Primary care patient cohort database (AUBMC); retrospective records of hospitalization and ED presentations	Polypharmacy was independently associated with significantly higher all-cause hospitalization (adjusted odds ratio [aOR] 1.66, 95% confidence interval [CI] 1.08–2.56, *p* = 0.022) and a trend toward more ED visits (40.6% vs. 31.4%, *p* = 0.05).
Cho et al. (2023) South Korea [1] Aim: To examine the national prevalence and clinical factors associated with polypharmacy and hyper-polypharmacy in the older population.	Nationwide population-based retrospective cohort South Korea; 12-month observation period	All Korean elderly ≥ 65 years covered by national health insurance *N* = 7,358,953	National Health Insurance Service (NHIS) claims database; prescription dispensing records (polypharmacy: ≥5 drugs ≥ 90 days; hyper-polypharmacy: ≥10 drugs)	The prevalence of polypharmacy was 47.8%, and that of hyper-polypharmacy was 11.9%, both of which were strongly associated with male sex, advanced age, multiple comorbidities, and frequent healthcare utilization.
Chau et al. (2025) Hong Kong [13] Aim: To evaluate the prevalence of potentially inappropriate medication (PIM) use and its association with the risk of hospitalization among older adults in residential care homes.	Prospective observational cohort 29 residential care homes for the elderly (RCHEs), Hong Kong Duration: 12 months (January–December 2023)	Older adults residing across 29 RCHEs *N* = 6346	SafeMed Medication Management System (SMMS^®^) records; PIM assessment using 2023 Beers criteria	The 12-month prevalence of PIMs was 34.5%. PIM use was independently associated with a higher hospitalization risk (OR 1.73, 95% CI 1.54–1.69), and the use of >1 PIM was associated with a significantly elevated risk (OR 2.17).

Note. aOR, adjusted odds ratio; AUBMC, American University of Beirut Medical Center; CKD, chronic kidney disease; ED, emergency department; HR, hazard ratio; KNOW-CKD, Korean Cohort Study for Outcomes in Patients with CKD; NHIS, National Health Insurance Service; OR, odds ratio; PAL-HF, Palliative Care in Heart Failure; PIM, potentially inappropriate medication; PPO, potential prescribing omission; RCHE, residential care home for the elderly; STOPP/START, Screening Tool of Older Persons’ Prescriptions/Screening Tool to Alert to Right Treatment.

**Table A2:** Observational and Cross-Sectional Studies (*n* = 7).

Study (Year) & Country/Study Aim	Design, Setting & Follow-Up Duration	Population & Sample (*N*)	Sampling Method & Data Source/Measurement	Key Findings (Primary & Outcomes)
Sallevelt et al. (2022) Multinational (OPERAM) [38] Aim: To assess the detectability of medication errors prior to potentially preventable drug-related hospital admissions in older adults with multimorbidity.	Observational diagnostic study nested in the OPERAM RCT intervention arm Multinational hospitals (4 European countries) Duration: Hospital readmission review period	Hospitalized older adults ≥ 70 years with multimorbidity and polypharmacy *N* = 963 (OPERAM intervention arm)	OPERAM intervention arm data; post-discharge readmission adjudication using STOPP/START criteria; retrospective medication error detectability analysis	8.7% experienced a potentially preventable drug-related readmission; 48% of medication errors at readmission were undetectable during the initial in-hospital review; of the detectable errors, ~25% received no recommendation, and ~25% of recommendations were not implemented.
Rogero-Blanco et al. (2021) Spain [14] Aim: To detect drug–drug interactions (DDIs) using a computer-assisted prescription system in multimorbid primary care patients.	Observational study using baseline data from the MULTIPAP RCT Primary care, Spain Duration: Cross-sectional baseline analysis	Multimorbid patients with ≥3 chronic conditions and polypharmacy (≥5 drugs) *N* = 480	MULTIPAP trial baseline prescription data; computer-assisted prescription software with integrated electronic DDI alert generation	DDIs were identified in 27% of patients, and 19% of high-risk regimens were modified following system alerts, demonstrating that automated electronic tools can produce clinically meaningful prescribing improvements at scale.
Oliveira et al. (2024) Portugal [10] Aim: To assess the prevalence of polypharmacy, DDIs, and severe DDIs in older patients with cancer receiving chemotherapy.	Cross-sectional observational 3 hospitals, northern Portugal Duration: Single time-point assessment	Older cancer patients ≥ 65 years receiving antineoplastic agents *N* = 552	Self-reported medication histories combined with medical record review; DDI classification using Micromedex^®^ software v 1.2.2	The prevalence of polypharmacy was 88.4%, and 76.45% of the patients had ≥ 1 DDI. Moreover, 56.16% of the patients had severe DDIs, and high-risk medications were independently associated with polypharmacy and DDI risk, underscoring the critical need for targeted reviews of oncological medications.
Abukhalil et al. (2022) Palestine [39] Aim: To evaluate the prevalence of potentially inappropriate prescribing (PIP) in hospitalized elderly patients using the STOPP/START criteria.	Retrospective descriptive chart review 2 major Palestinian hospitals Duration: Retrospective study period	Hospitalized elderly patients ≥ 65 years *N* = 247	Inpatient medical record chart audits using STOPP/START criteria for PIM detection and classification	PIP prevalence was 66.8% among hospitalized older adults, and polypharmacy was observed in 44.5% of patients. The STOPP/START criteria demonstrate high clinical detection utility in resource-limited contexts.
Lopez-Rodriguez et al. (2020) Spain [9] Aim: To detect potentially inappropriate prescriptions in multimorbid primary care patients using explicit (STOPP) and implicit (MAI) criteria.	Cross-sectional baseline analysis of the MULTIPAP trial Primary care, Spain Duration: Cross-sectional	Community-dwelling older adults aged 65–74 years with multimorbidity (≥3 diseases) and polypharmacy *N* = 593	MULTIPAP trial baseline prescription records; assessed using STOPP criteria (explicit) and the Medication Appropriateness Index/MAI (implicit) in parallel	High rates of inappropriate prescribing were detected by both tools, and explicit and implicit criteria identified complementary, non-overlapping PIPs, confirming the clinical value of combining both approaches to maximize the detection.
Ferrari et al. (2018) Italy [40] Aim: To evaluate the prevalence, characteristics, and risk factors of polypharmacy in patients with primary headache disorders.	Cross-sectional clinical study Specialized headache center, Italy Duration: Single-time-point assessment	Patients with primary headache disorders *N* = 300	Specialized clinical records and complete medication histories from a consecutive clinic sample	Polypharmacy was observed in 40.7% of patients overall and 58.8% of patients with chronic headache. It was significantly associated with comorbidities, prophylactic treatment, and triptan use (*p* < 0.001).
Dobrică et al. (2019) Romania [8] Aim: To evaluate and compare polypharmacy patterns and drug–drug interactions in patients with type 2 diabetes mellitus patients vs. non-diabetic controls.	Cross-sectional retrospective observational Internal medicine ward, Clinical Emergency Hospital of Bucharest, Romania Duration: 2-month	Hospitalized patients *N* = 126 (63 type 2 diabetes mellitus; 63 matched controls)	Inpatient medical records review; DDI assessment from documented prescriptions during a 2-month review period	Patients with diabetes were prescribed significantly more drugs (7.81 ± 2.23 vs. 5.33 ± 2.63, *p* = 0.0001) and had significantly more DDIs (8.86 ± 5.76 vs. 4.98 ± 5.04, *p* = 0.0003), demonstrating the compounded pharmacological burden in T2DM.

Note. DDI, drug–drug interaction; MAI, Medication Appropriateness Index; PIM, potentially inappropriate medication; PIP, potentially inappropriate prescription; STOPP/START, Screening Tool of Older Persons’ Prescriptions/Screening Tool to Alert to Right Treatment.

**Table A3:** Qualitative Studies (*n* = 7).

Study (Year) & Country/Study Aim	Design, Setting & Follow-Up Duration	Population & Sample (*N*)	Sampling Method & Data Collection	Key Findings (Primary & Outcomes)
Zhai et al. (2022) China [17] Aim: To identify barriers and facilitators to implementing a nursing clinical decision support system (CDSS) in a tertiary hospital using the FITT framework.	Qualitative evaluation using FITT framework 4 medical–surgical wards, 2000-bed tertiary hospital, Shanghai Duration: Observational and interview periods	Hospital nurses and implementation stakeholders 200 h of participatory observation + 21 semi-structured interviews	Purposive sampling; participatory observation combined with semi-structured interviews; data analyzed within FITT (Fit between Individuals, Task, Technology) framework	12 core implementation categories identified; administrative leadership and operational support were key facilitators; user-interface design friction and inadequate training were primary barriers; management oversight identified as crucial for sustainable CDSS adoption.
Zechmann et al. (2019) Switzerland [6] Aim: To explore barriers and enablers affecting deprescribing practices among older multimorbid patients with polypharmacy in primary care settings.	Explorative qualitative study Primary care, Northern Switzerland Duration: Interview study	Older multimorbid patients with polypharmacy (≥5 medications) *N* = 19	Convenience sampling; semi-structured telephone interviews; thematic analysis	Patient trust in GP was the primary facilitator of deprescribing, and the main barriers included clinical inertia, fragmented care, and fear of symptom relapse. No participant felt devalued by the deprescribing discussion.
Schmidt-Mende et al. (2018) Sweden [15] Aim: To understand general practitioners (GP and nurses’ perspectives on medication reviews and the management of potentially inappropriate medicines in older patients with polypharmacy.	Qualitative study nested within a cluster RCT 33 primary care practices, Stockholm, Sweden Duration: Throughout the trial period	GPs (*N* = 194) and clinical nurses (*N* = 113) From 33 primary care practices	Purposive sampling from 33 practices; narrative and unstructured reflective diaries from pharmacists and clinicians collected during the trial	Five key themes were identified: clinical complexity of aging, conflict with prescribing guidelines, real-world constraints, multistep nature of medication review, and perceived threats to GP clinical autonomy. Reviews were valued but were routinely deprioritized under the time pressure.
Rezahi et al. (2021) Canada [25] Aim: To explore the clinical roles and contributions of pharmacists in primary care team-based medication reviews.	Qualitative content analysis Family Health Teams (FHTs), Ontario, Canada Duration: Survey study	FHT clinical pharmacists *N* not specified in original publication	Convenience sampling; structured open-ended survey responses; qualitative content analysis	Core functions included direct medication management and patient counseling/education, with diabetes, cardiovascular disease, and chronic pain management as the primary clinical targets, confirming the broad contribution of pharmacists to team-based care.
Little et al. (2023) USA [28] Aim: To elicit perspectives from long-term care staff, residents, and family proxies regarding deprescribing practices and to identify actionable implementation steps.	Qualitative thematic analysis Three long-term care facilities, USA Duration: Interview study	Nurses, nurse aides, nurse practitioners, residents, and family proxies *N* = 28 participants (26 semi-structured interviews)	Purposive sampling; semi-structured interviews; thematic analysis	Three overarching themes were identified: establishing patient–provider trust, identifying motivating factors for deprescribing, and standardizing supportive institutional processes. Six actionable clinical steps for sustainable LTC deprescribing were outlined.
Lee et al. (2023) USA [16] Aim: To understand the perspectives of healthcare workers, patients, and caregivers on establishing a deprescribing program in the emergency department.	Thematic qualitative analysis Academic emergency department, USA Duration: Interview and focus group study	ED healthcare workers, older patients, and caregivers *N* = 28 (8 focus groups; 20 individual interviews)	Purposive sampling; focus groups combined with semi-structured individual interviews; thematic analysis	Four main themes were identified: challenges in obtaining accurate medication histories, missed opportunities for intervention, system-level barriers (time pressure, staffing), and the need for coordinated discharge recommendations.
Brünn et al. (2021) Germany [35] Aim: To explore patient views on polypharmacy and digital clinical decision support systems for general practitioners (GPs) nested within the AdAM cluster randomized controlled trial (RCT).	Thematic qualitative analysis nested in a cluster RCT General practice clinics, Germany Duration: During cluster RCT participation	Older adults with polypharmacy *N* = 13 (in-depth interviews)	Purposive sampling from AdAM cRCT; in-depth semi-structured interviews; thematic analysis	Seven clinical themes were identified: patients generally accepted polypharmacy out of habit, maintained high trust in general practitioners (GPs), and viewed digital medication safety support tools favorably as a complement to physician clinical oversight.

Note. CDSS, clinical decision support system; ED, emergency department; FITT, Fit between Individuals, Task, and Technology; FHT, Family Health Team; LTC, long-term care.

**Table A4:** Descriptive, Comparative, Mixed-Methods, Validation, and Conceptual Studies (*n* = 6).

Study (Year) & Country/Study Aim	Design, Setting & Follow-Up Duration	Population & Sample (*N*)	Sampling Method & Data Source/Measurement	Key Findings (Primary & Outcomes)
Thevelin et al. (2019) Belgium [19] Aim: To compare the diagnostic sensitivity of the STOPP/START criteria versions 1 and 2 in detecting PIMs and PPOs before geriatric hospital admission.	Comparative diagnostic analysis Geriatric hospital, Belgium Duration: Retrospective chart analysis	Geriatric hospitalized patients *N* not specified in original publication	Head-to-head application of STOPP/START v1 and v2 to the same patient records; comparison of detection rates for PIMs and PPOs	STOPP/START version 2 increased the detection of PIMs and PPOs from 23% (v1) to 40% (v2). The updated criteria substantially improved the identification of clinically relevant drug-related errors, and v2 is recommended for routine clinical use.
Lea et al. (2019) Norway [3] Aim: To determine the prevalence of drug-related hospitalizations (DRHs) and associated risk factors in multimorbid internal medicine inpatients.	Descriptive observational study Internal medicine ward, Norway Duration: Hospital admission period	Multimorbid internal medicine inpatients *N* = 450	Detailed chart review extracted from cluster-RCT clinical trial records; multivariate risk factor analysis	Polypharmacy and inappropriate prescribing have been identified as the primary contributors to preventable DRHs, with estimated rate of preventable DRHs ranging from 10% to 30%, confirming a significant preventable burden.
Snijders et al. (2023) Netherlands [41] Aim: To validate and update the OPERAM drug-related admission (DRA) prediction tool for older hospitalized adults.	External validation study Hospital datasets, Netherlands Duration: MedBridge trial cohort validation	Older hospitalized adults ≥ 65 years with multimorbidity and polypharmacy *N* = 2516 (MedBridge cohort)	Statistical re-calibration and external validation using the MedBridge trial cohort; C-index discrimination and Hosmer–Lemeshow calibration assessment	Removing the hepatic cirrhosis predictor and adding the active polypharmacy variable improved model discrimination (C-index 0.64 vs. 0.62) and calibration, enhancing the clinical utility for identifying high-risk patients requiring targeted pharmacist-led reviews.
Mercer et al. (2018) Canada [5] Aim: To examine how physicians and pharmacists communicate medication information and how this influences EHR design for interdisciplinary medication management.	Mixed-methods evaluation (semi-structured interviews + direct clinical observation) 19 pharmacies and 9 medical clinics, 4 Canadian provinces Duration: Data collection period	34 healthcare professionals (physicians and pharmacists)	Direct clinical observation combined with semi-structured interviews across community pharmacies and team-based primary care clinics in 4 provinces	Limited interdisciplinary communication was observed; pharmacists frequently lacked key prescribing information (indication, adherence history); current EHR architectures do not facilitate collaborative medication management; user-centered EHR redesign is strongly recommended.
Băjenaru et al. (2024) Romania [36] Aim: To discuss geriatric healthcare supported by digital decision-making tools integrated into the RO-SmartAgeing and NeuroPredict digital health platforms.	Descriptive health technology evaluation Geriatric care, Romania Duration: Not applicable (descriptive)	Not applicable (digital health technology description and evaluation)	Technical review of integrated digital health monitoring platforms (Medical Blackbox, Gaitband, RO-SmartAgeing, NeuroPredict); narrative evaluation framework	The deployment of specialized digital tools within integrated platforms improved clinical oversight and medication safety monitoring for older adults, highlighting the critical need for clinician–AI collaboration and sustained governance frameworks.
Sopruchi et al. (2024) International [37] Aim: To examine the potential, challenges, and future directions of integrating AI-driven clinical decision support systems into routine healthcare practice.	Conceptual analysis General healthcare context Duration: Not applicable (conceptual)	Not applicable (narrative literature synthesis on AI-DSS frameworks)	Narrative review of AI health technology frameworks; integrative evaluation of implementation evidence and governance literature	Machine learning algorithms can process complex multimorbid datasets; however, their clinical deployment is constrained by data quality concerns, algorithmic bias, limited interpretability, and low practitioner trust. A responsible AI-DSS integration framework was proposed.

Note. AI, artificial intelligence; AI-DSS, artificial intelligence-based decision support system; DRA, drug-related admission; DRH, drug-related hospitalization; EHR, electronic health record; PIM, potentially inappropriate medication; PPO, potential prescribing omission; STOPP/START, Screening Tool of Older Persons’ Prescriptions/Screening Tool to Alert to Right Treatment.
